# Subjective memory complaints associated with depression and cognitive impairment in the elderly: A systematic review

**DOI:** 10.1590/S1980-57642015DN91000009

**Published:** 2015

**Authors:** Allan Gustavo Brigola, Carlene Souza Silva Manzini, Gabriel Brassi Silveira Oliveira, Ana Carolina Ottaviani, Michelli Pacheco Sako, Francisco Assis Carvalho Vale

**Affiliations:** 1 Bacharel em Gerontologia, Mestrando no Programa de Pós-Graduação em Enfermagem da Universidade Federal de São Carlos.; 2 Enfermeiro, Mestrando no Programa de Pós-Graduação em Enfermagem da Universidade Federal de São Carlos.; 3 Médico Neurologista, Professor de Medicina e docente do Programa de Pós-Graduação em Enfermagem, da Universidade Federal de São Carlos.

**Keywords:** aged, memory, dementia, depression, anxiety

## Abstract

The aging process can be accompanied by a slight decline in cognitive functioning, and subjective memory complaints (SMC) appear to be common in the elderly population.

**Objective:**

To determine whether SMC is associated with cognitive loss or depression and can predict dementia.

**Methods:**

A systematic review of the literature was conducted. Articles were selected on the following databases, LILACS, SCOPUS, SCiELO, PubMed and Web of Science from August to October 2013. Article selection was based on inclusion and exclusion criteria. Studies published between 2010 and 2013, written in English, Spanish or Portuguese, involving populations 65 years or older, were included. Reviews were excluded.

**Results:**

After the selection, a summary of the 20 articles retrieved was carried out. Of the total articles retrieved, fifteen were cross-sectional studies and five were longitudinal studies. Most of the cross-sectional studies associated SMC with depression, objective cognitive impairment and anxiety. The emergence of dementia in people with SMC was evidenced in longitudinal studies. Albeit less frequently, SMC were also associated with reduced quality of life, impairment in Activities of Daily Living (ADL), emergence of neuropsychiatric symptoms, lower hippocampal volume, amygdala volume reduction, increased activation of the left temporal, bilateral thalamus, caudate and posterior cingulate, and with the occurrence of ApoE ε4.

**Conclusion:**

SMC may be associated with changes in mood and/or cognition, and its occurrence appears to increase the likelihood of dementia. In order to further our understanding of the topic, future studies should consider the recruitment of representative samples with control groups and longitudinal designs.

## Abstract (pt)

O processo de envelhecimento pode vir acompanhado de um declínio leve no funcionamento cognitivo e a queixa subjetiva de memória (QSM) parece ser frequente na população idosa.

**Objetivo:**

Verificar se a QSM está associada à perda cognitiva ou depressão e se pode predizer demência.

**Métodos:**

Uma revisão sistemática da literatura foi realizada. Artigos foram selecionados nas seguintes bases de dados LILACS, SCOPUS, SciELO, PubMed e *Web of Science* entre agosto e outubro de 2013. Para seleção dos artigos foram usados critérios de inclusão e exclusão. Foram incluídos estudos publicados entre 2010 e 2013, escritos em inglês, espanhol ou português, com amostras de pessoas com 65 anos ou mais. Revisões foram excluídas.

**Resultados:**

Após a seleção, uma síntese foi feita de 20 artigos. Quinze eram estudos transversais e cinco estudos longitudinais. A maioria dos estudos transversais associaram QSM com depressão, comprometimento cognitivo objetivo e ansiedade. A ocorrência de demência em pessoas com QSM foi evidenciado nos estudos longitudinais. Com menor frequência, QSM estava associada a redução da qualidade de vida, prejuízo nas Atividades da Vida Diária (AVD), surgimento de sintomas neuropsiquiátricos, menor volume hipocampal, redução do volume da amígdala, maior ativação do lobo temporal esquerdo, tálamo bilateral, cingulado posterior e caudado, e ocorrência de ApoE ε4.

**Conclusão:**

QSM pode estar associada a mudanças no humor e/ou cognitivas, e sua ocorrência parece aumentar a chance para demência. Para contribuir com o entendimento do assunto, futuras investigações podem considerar o recrutamento de amostras representativas, com grupos controles em desenhos longitudinais.

## INTRODUCTION

The aging process can be accompanied by a slight decline in cognitive functioning, and subjective memory complaints (SMC) is a common symptom in the elderly population. SMC is also described in the literature as "reports of memory loss", "subjective memory problems", "subjective cognitive loss" or simply "memory complaints".

Cognitive function is a major determinant factor in assessments of the quality of life in senescence, and its decline contributes to increasing elderly dependence.^1^ Cognitive impairment in elderly can be diagnosed based on a detailed history and cognitive examination using various instruments. These instruments aim to obtain information that supports both the syndromic and etiological diagnosis and the planning and execution of therapeutic and rehabilitation measures to be used in each case.^2^ SMC is a common symptom in adults whose prevalence increases with age. A review of previous studies based on the elderly population shows a prevalence of 46.3% among adults 50-59 years old and of 63.4% in elderly patients 80-100 years old.^3^ Female sex and low educational level have also been associated with a higher prevalence of SMC.

A number of instruments for evaluating SMC are described in the literature, such as the Memory Complaint Scale (MCS), designed for active search of memory complaints from both the subject and a companion who knows them well,^4^ the Assessment of Memory Complaint (MAC-Q), which assesses age-related memory decline^5^ and the Informant Questionnaire on Cognitive Decline in the Elderly (IQCODE), a screening instrument with 26 questions collecting information provided by family members or caregivers on possible patient cognitive decline.^6^

Several studies have shown that older adults with depressive symptoms had significantly more SMC compared to older adults without these symptoms. Thus, depressive symptoms appear to be important predictors of SMC.^7-9^

The aim of this review was to investigate whether SMC is associated with cognitive impairment or depression and can predict dementia. This review is justified by the need to broaden understanding on variables related to SMC, considering their relevance in guiding the diagnosis in cognitive and mood disorders.

## METHODS

This study consists of a systematic literature review. The search for scientific papers was performed between August and October, 2013, and used LILACS, SCOPUS, Web of Science, PsycINFO, PubMed and SciELO databases.

The descriptors for the search were obtained in MeSH and DeCS. Additionally, the terms subjective memory complaint and memory complaint were used as, although not indexed, they are frequently used in the literature. The following operations were performed on the databases: (subjective memory complaint), (elderly AND subjective memory complaint) and (elderly AND memory complaint). In addition, the operations (elderly AND subjective memory complaint AND dementia), and (elderly AND memory complaint AND dementia) were also used. In the search, the more restrictive operations retrieved items not found in the broader operations.

For selection of the articles retrieved by at least two reviewers, the following inclusion criteria were used: publication in peer-reviewed journals between 2010 and 2013 in English, Spanish or Portuguese, available in full and studying populations aged 65 years old or older. Review articles were excluded.

## RESULTS

**Figure 1:**
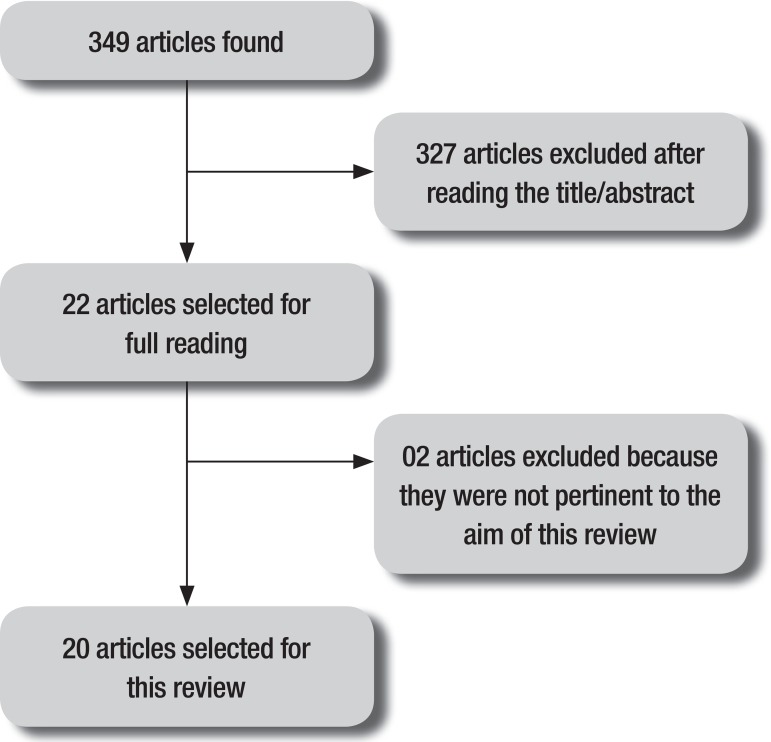
Illustrative summary of articles for review selection.

A summary of the methods used and the findings is given in Figure 1. From the raw number of articles identified in the database (n=349), a total of 20 studies were selected for this review.

Of the articles used in this review, 15 were cross-sectional studies and 5 longitudinal studies. Tables 1 and 2 summarize the information from these groups of studies, respectively.

**Table 1:** Cross-sectional studies on subjective memory complaints.

Author (year)	Place	Population Base	n	Age	SMC associated with
Balash et al. (2013)	Tel Aviv, Israel	Population-based/Epidemiological	636	68*	Depression/anxiety
Kim et al. (2013)	Seoul, South Korea	Casuistry/Case-control	118	>65.8	Hippocampal volume decrease/depression/dementia
Montejo et al. (2012)	Madrid, Spain	Population-based/Epidemiological	1637	>64	Quality of life/ADL
Vale, Balieiro-Junior and Silva-Filho (2012)	Ribeirão Preto and Manaus, Brazil	Casuistry^##^	161	>60	Depression/Deterioration in cognitive status/dementia
Pires et al. (2012)	Lisbon, Portugal	Population-based/Epidemiological	871	≥50	MCI/deterioration in cognitive status
Amariglio et al. (2011)	Boston, USA	Population-based/Epidemiological	16964	70-81	Deterioration in cognitive status
Bucley et al. (2011)	Melbourne, Australia	Casuistry/Case-control	740	72-78	Depression/anxiety
Hurt et al. (2011)	Manchester, England	Casuistry^##^	98	73.4*	Depression/anxiety
Mascherek et al. (2011)	Nuremberg, Germany	Casuistry/Case-control	169	76.24*	Deterioration in cognitive status**
Aguiar, Ribeiro and Jacinto (2010).	Sao Paulo, Brazil	Casuistry/Case-control	28	75-81	Unassociated
Dujardin et al. (2010)	Lille, France	Population-based/Epidemiological	180	62*	Deterioration in cognitive status/dementia
Fischer et al. (2010)	Toronto, Canada	Casuistry^##^	85	≥50	Depression/socioeconomic status
Paulo, Yassuda (2010)	São Paulo, Brazil	Casuistry^##^	67	60-75	Anxiety
Rodda et al. (2010)	London, England	Casuistry/Case-control	11	64.6*	Increased activation in left medial temporal lobe, bilateral thalamus, posterior cingulate and caudate
Tournier, Postal (2010)	Bordeaux, France	Population-based/Case-control	28	68.5*	Depression^#^

* Average age;

** Language;

# Associated with metamemory;

## Not specified. ADL: Activities of Daily Living; MCI: Mild Cognitive Impairment.

**Table 2:** Longitudinal studies on subjective memory complaints.

Authors (year)	Place	Population base	n	Follow-up (years)	Age	Cognitive status	Evolving to
Chary et al. (2013)	Bordeaux, France	Population-base/Cohort	2882*	20	≥65	MMSE 26.5	Dementia
Heser et al. (2013)	Hamburg, Leipzig Mannheim, Munich, Germany	Population-based/Cohort	2663*	1.5	81.2^#^	–	Alzheimer Disease
Waldorff et al. (2012)	Copenhagen, Denmark	Population-based/Cohort	758*	4	≥65	MMSE 28.2	Dementia
Stewart et al. (2011)	Bordeaux, Dijon, Montpellier, France	Population-based/Cohort	1336*	4	≥65	MMSE 27.6	Hippocampal Volume Decrease/Deterioration in cognitive status
Gallassi et al. (2010)	Bologne, Italy	Casuistry/Incidence	92**	4	–	MMSE ≥23.8	Dementia/MCI/Deterioration in cognitive status

* Community sample;

** Reference sample;

# Average age; MMSE: Mini-mental State Examination; MCI: Mild Cognitive Impairment.

A study conducted by Fischer et al. (2010)^10^ in users of a memory clinic aged from 50 years old, sought to track factors associated with SMC. A significant relationship between frequent memory complaints and depression as well as educational level was found. Similar results were found in another study by Tournier and Postal (2011)^11^ in highly educated elderly and adults with Mini-Mental State Examination (MMSE) scores > 26 and no history of neurological diseases, in which depressive symptoms influenced metamemory both in adults and in the elderly. In order to compensate for this neglect, the elderly used more external strategies (listing names, dates, phone numbers, to use schedules) than young adults.

SMC was also associated with depression in other studies such as those by Balash et al. (2013)^12^ and Buckley et al. (2013).^13^ Comparing the two 2013 studies, one involved a sample of 636 elderly, mostly women (61%) and concluded that the presence of SMC was not associated with the objective cognitive performance assessed by Mindstreams, a cognitive battery, but was associated with MMSE scores, and was also associated with symptoms of depression and anxiety in cognitively normal elderly.^12^ Affective variables were associated with SMC severity in the elderly cognitively healthy group, whereas in the Mild Cognitive Impairment (MCI) group SMC severity was associated with age. In both groups, SMC was not associated with cognitive variables or biomarkers for AD (ApoE ε4).^13^

Another finding was reported by Hurt et al. (2011)^14^ in a sample of 98 elderly with average age of 73 years, where SMC was associated with depression and anxiety in the oldest patients. On the other hand, a survey conducted in the city of São Paulo, Brazil, by Paulo and Yassuda (2010)^15^ in 67 seniors from a social interaction environment without cognitive impairment according to the MMSE and having scores ≤ 5 on the Geriatric Depression Scale (GDS), found that the frequency of forgetfulness episodes was related only to anxiety symptoms and also identified an association between anxiety symptoms and depression symptoms. The depression, cognition and education variables were not associated directly with SMC.

Vale, Balieiro-Junior and Silva-Filho (2012),^4^ who conducted another Brazilian study in the cities of Manaus and Ribeirao Preto, proposed the Memory Complaint Scale (MCS) as a tool for active search of memory complaints in two ways: directly with the patient and also through a companion who knows the patient well. One hundred and sixty-one seniors and family members were interviewed, scoring 0-3 on the Clinical Dementia Rating (CDR). The answers on the MCS given by the senior and the family were associated with MMSE performance, whereas only the answers given by the elderly were associated with depression symptoms. Another important result was that the higher the CDR, the lower the memory complaints reported by the elderly (inverse relationship with dementia) and the higher by the family (direct relationship with dementia).

The case-control study of Mascherek et al. (2012)^16^ in 169 elderly from a specialized clinic with an average age of 76 years aimed to examine whether cognitive complaints were differentially related to cognitive functioning in different groups. Multiple regression analyses revealed that, after controlling for depression, age, sex and education, global cognitive performance was not associated with cognitive complaints.^16^

Aguiar, Ribeiro and Jacinto (2010)^17^ conducted a controlled study using the protocol for assessment of global health that included the Mini-Mental State Examination (MMSE), the Dementia Rating Scale Mattis (Mattis-DRS) plus a question about memory complaints ("how good is your memory?"). To determine whether there was a correlation between SMC and cognitive decline, they correlated the scores obtained on each of the items of the instruments with the presence of SMC between two groups of elderly. The first group comprised individuals with SMC living in the community and the second comprised residents from an institution for the aged without SMC, with average ages of 81 and 75 years, respectively. The results showed no statistically significant difference between the two groups for scores on the cognitive tests suggesting that SMC might be associated with extrinsic factors other than insipient cognitive decline.^17^

In another study, measures of objective cognitive status had a direct and close relationship with SMC, and also showed that forgetting things rapidly (seconds) can be generally associated with aging.^18^ Tests involving telephone-based cognitive assessments and seven questions regarding SMC were administered. In fact, there were many trends of increasingly worse scores on cognitive tests with higher number of memory complaints. Amariglio et al. (2011)^18^ suggested the use of more detailed evaluations in suspected cases of Alzheimer Disease (AD).

Dujardin et al. (2010)^19^ found that objective cognitive decline was associated with SMC in a sample of patients with Parkinson's disease. A total of 180 patients were evaluated, 55.55% of whom were men with a mean age of 62 years old with 11 years of schooling. The results revealed that the frequency of objective cognitive decline was significantly greater in patients with SMC.

Cognitive status was not associated with the SMC in the study carried out in Portugal by Pires, Schmand and Silva (2012)^20^ assessing 871 subjects without dementia or other neurological disorders and MMSE scores ≥23, comprising 581 from the community and 290 who sought medical assistance. However, all clinical participants recruited had at least one SMC, the most frequently reported being forgetting names of friends and family members.

Kim et al. (2013)^21^ conducted a specific research in a study conducted in South Korea whose purpose was to ascertain whether individuals with SMC had lower hippocampus or amygdala volume compared to the control group, and whether their depressive symptoms were associated with these differences in volume. Subjects with SMC had lower volumes in the hippocampus and amygdala and consequently more depressive symptoms (average 12.5 symptoms) compared with individuals without SMC. In subjects with SMC, depressive symptoms were directly associated with hippocampal volume reduction. A similar finding was demonstrated in another study of community-dwelling elderly residents with SMC performed by Stewart et al. (2012).^22^ Neuroimaging (MRI) was used and repeated over four years. The results found that changes in hippocampal volume, total gray matter and cerebrospinal fluid volume, besides increase in sub-cortical white matter lesions, were associated with SMC, independently of the presence of cognitive decline or depressive symptoms. Moreover, depressive symptoms were more evident in individuals with genotype to ApoE ε4.^22^

In a British study, elderly divided into a control group with no SMC and a group with SMC, without MCI or dementia, underwent neuroimaging and functional magnetic resonance imaging (fMRI) exams. Rodda et al. (2011)^23^ demonstrated greater activation in the left medial temporal lobe, bilateral thalamus, as well as the posterior and caudate cingulate in the SMC group compared to the control group. The authors concluded that compensations in brain function (plasticity) occur in individuals with SMC.

The emergence of dementia in subjects with SMC was evidenced in longitudinal studies (Table 2). A study conducted in France sought to distinguish short and long term predictors for dementia according to educational level in 2882 subjects followed for 20 years. Based on the results, Chary et al. (2012)^24^ concluded that IADL dependencies can predict dementia in elderly with low education and SMC in elderly patients with high education.

In older adults aged over 75, Heser et al. (2013)^25^ found that depression was a prodromal state for AD but not for dementias of other etiologies. The authors suggested that clinicians take into account the parameters of depression and subjective memory loss, as these may predict dementia, independently of the presence of cognitive decline.^25^

A study of hospitalized elderly patients with a mean age of 74.8 years spanning four years of follow-up, showed that SMC were independent predictors for a dementia diagnosis. Other risk factors were MMSE <24, age from 75 to 84 years and need for help performing IADL. Waldorff et al. (2013)^26^ also concluded that SMC should be used to identify vulnerable elderly.

Gallassi et al. (2010)^27^ conducted a longitudinal study in 92 outpatients with subjective cognitive complaints (49 with MCI and 43 with cognitive impairment only) were interviewed over a four-year period. In the sample, there were no significant differences in assessed measures of depressive symptoms in subjects with subjective cognitive complaints compared to the group of cognitively normal subjects. At the end of follow-up, for the group with MCI, 32.2% were stable, 18.4% had developed dementia and 4% had reverted to cognitive impairment only. In the group with cognitive impairment only, 45.5% were unchanged, 13.9% evolved to MCI and only one subject progressed to dementia.

In another study by Montejo et al. (2012)^28^ in a sample of 1637 non-institutionalized elderly, SMC was associated with lower quality of life. In elderly with more limitations on social activities, memory complaints occurred at a frequency of 72.9%. Among the participants dependent for IADL, 52% presented SMC, while only 25.7% of those independent for IADL reported memory complaints. Among the IADL, SMC occurred more frequently in those who had difficulty using the telephone, managing medications and dealing with finances.^28^

**Figure 2:**
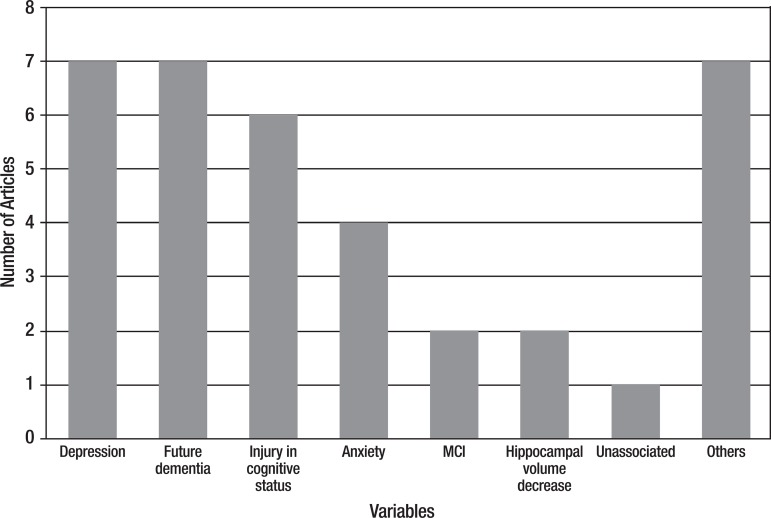
Variables associated with SMC

Figure 2 shows the frequency of variables associated with SMC. The most frequently associated independent variables were depression, predicted dementia and cognitive decline.

SMC was also associated with the following variables individually: reduced quality of life, ADL impairment, neuropsychiatric symptoms, reduced amygdala volume, activation of the left temporal lobe, bilateral thalamus, caudate and posterior cingulate, ApoE ε4 occurrence and socioeconomic status. These variables were grouped, and placed in the figure, under the "Others" category.

## DISCUSSION

There is a considerable number of studies on SMC in the elderly. However, defining SMC as early indicators of specific disorders, such dementia, is complex and difficult. Previous reviews have attempted to establish this relationship. Jonker, Geerlings and Schmand, in a summary of ten population-based studies, showed that SMC can be associated with cognitive impairment and can predict dementia in a two-year follow-up. Following the same line of reasoning, Roberts, Clare and Woods, in a review of sixteen studies, showed that awareness can vary and predicts dementia.^29^ On the other hand, Riedel-Heller et al. speculated that memory complaints cannot be taken as a clear clinical indicator for cognitive impairment and may reflect depressive disorders and a multitude of other processes, of which an objective cognitive impairment is just one aspect.^30^

To contribute to our understanding on the topic, future studies should consider the recruitment of representative samples with control groups using longitudinal designs. It would be advisable to search for biomarkers and the use of appropriate measurement instruments (e.g. neuroimaging exams have strongly contributed to the evaluations) in order to confer increased reliability to the results.

Despite the relatively small number of articles retrieved, this review revealed, as shown in Figure 2, that depression and future occurrence of dementia ranked equal. Also, it is important to note that objective cognitive impairment and anxiety can be part of a scenario in which the memory complaint is a symptom.

Further reviews should be carried out with differentials, including meta-analysis and integrative reviews. Currently, there are several review protocols that can assist in performing methods and summarizing results. The opinion of specialists is important in consolidating the results of a review and the uniqueness of each sample should be respected.

To conclude, subjective memory complaints are common in the elderly population. This is better evidenced if an active search with the patient is conducted and also when the search includes information on a companion who knows the patient well. At the time of study publication, the scientific literature provided no definition of the nature and cause of SMC. The studies considered in this review highlight depression, future dementia and cognitive impairment as the factors most often associated with this complaint. Notwithstanding its undefined etiology, SMC should not be taken as only a trivial symptom. It warrants the attention of health professionals and careful clinical investigation, as it may signal current mood or cognition alterations and the future occurrence of dementia.
